# Unequal burden of equal risk factors of diabetes between different gender in India: a cross-sectional analysis

**DOI:** 10.1038/s41598-021-02012-9

**Published:** 2021-11-22

**Authors:** Sujata, Ramna Thakur

**Affiliations:** School of Humanities and Social Sciences, Indian Institute of Technology Mandi, Kamand, Himachal Pradesh 175075 India

**Keywords:** Health care economics, Health policy, Public health

## Abstract

Many studies have supported that the burden of diabetes is shared differently by different genders due to various factors associated with it. This study aims at capturing whether women and men with a similar background, dietary and smoking habits, and biological conditions (blood pressure and body mass index (BMI)) are being affected equally or differently by diabetes. We have used cross-sectional data of NFHS-4 by covering the age group 15–49 years. Association between socio-economic background, dietary habits, biological conditions, and diabetes has been estimated using two separate multivariate logistic regression models. Results show that the overall prevalence of diabetes is higher among men (2.63%) than women (2.35%). Whereas, women belonging to urban areas (3.53%), Christian category (3.92%), richer section (3.22%), women with no schooling (2.51%), those reported never to consume pulses (2.66%) and green vegetables (2.40%) and daily consuming eggs (3.66%) and chicken or meat (3.54%) are more affected by diabetes than their men counterparts. Whereas men residing in rural areas (2.30%), belonging to the general category (3.12%), SCs (2.37%) and STs (1.72%) are more affected than their women counterparts. Results have also shown a higher prevalence of diabetes among obese men (11.46%), non-vegetarian (2.71%) and those who watch television almost every day (3.03%) as compared to their women counterparts. Regression analyses show that the richest, hypertensive, and obese women and men are significantly more likely to suffer from diabetes. This study concludes that women and men with similar socio-economic status, biological conditions, dietary and smoking habits are being affected differently by diabetes. Thus, there is a need for gender dimension in research to understand and validate the differences in the needed interventions for diabetes control in India.

## Introduction

Diabetes is emerging as one of the significant causes of morbidity and mortality, mainly affecting adults and the middle-aged population in the world. It is one of the fastest-growing global health emergencies of the 21st century. Four hundred sixty-three million people have diabetes which is expected to increase up to 578 million by 2030 and 700 million by 2045^1^. WHO reports, the prevalence of diabetes among adults (aged 18 years and above) rose from 4.7% in 1980 to 8.5% in 2014 and premature mortality due to diabetes increased by 5% between 2000 and 2016. The report also says that the prevalence of diabetes is rising more rapidly in low-and-middle-income countries than in high-income countries^2^.

Many studies have pointed towards the sex and gender differences in epidemiology in recent years, particularly in diabetes^3^. Some studies have found a significant difference in the prevalence of diabetes among men and women. They argued that several biological, sociocultural and psychosocial (economic, environmental and behavioural) risk factors bring this gender difference^4,5^. As per the findings of some other studies, diabetes is more common among middle-aged men than women^6,7^. Another study supported this finding in Sweden that reported a 14.6% prevalence rate among men and 9.1% among women^8^.

It is reported in the literature that Indians develop diabetes at an earlier age than Western people and are also very likely to develop diabetes even with a very slight weight gain^9–11^. According to International Diabetes Federation (IDF) report, India stands in second place with more than 77 million diabetic people, and its contribution to regional (South East Asia) mortality is maximum^1^. Several factors contribute to the rapid rise in diabetic cases in the country. Some are industrialisation, urbanisation, dietary pattern, physical inactivity, alcohol and tobacco consumption and socio-economic conditions. With the narrowing gap in urban–rural living conditions and rapid socio-economic changes in rural areas, the prevalence of diabetes is likely to increase, which will further cause early morbidity and mortality among people in India^12,13^.

Studies conducted in India show conflicting results on the gender distribution of diabetes. Some reflections from north India show that females are more affected by diabetes, while other studies from southern India have shown a higher prevalence among males^14,15^. Several other studies have also demonstrated variations in the prevalence of diabetes by sex and concluded that men are more affected by diabetes than women in the country^16,17^. Studies have analysed socio-economic inequality in the prevalence of diabetes but left the gender differential analysis in its prevalence across different socio-economic groups^18,19^. Few other studies have focused either on a single state or the geographical variation in the prevalence of diabetes in the country^20–22^.

Further, a study has presented a comprehensive analysis of the burden of diabetes among Indian states from 1990 to 2016 but did not focus on gender dimension^23^. Studies have also shown an association of diabetes with the types of food and legume consumption^24,25^. Few studies have looked into gender differences in the prevalence of diabetes. Still, these studies have either focused on a single state of the country or have not captured some critical factors such as hypertension, body mass index, vegetarian status and frequency of watching television which also affect the occurrence of diabetes among different genders^17,26^. After seeing the above literature on diabetes, we found a lack of studies focused exclusively on the role of socio-economic and risk factors in the incidence of diabetes separately among men and women in India.

## Objective

Hence, the primary objective of this study is to analyse the role of socio-economic factors, dietary patterns, lifestyle and biological conditions (blood pressure and body mass index (BMI)) in the incidence of diabetes among different gender in India.

## Data and methods

The analysis is based on the fourth round of the National Family Health Survey (NFHS-4) conducted in all the twenty nine states and six union territories of India from 20th January 2015 to 4th December 2016. This survey included women and men aged 15–49 years and 15–54 years by taking sample sizes 699,686 and 112,122, respectively. The survey collected information on variables like socio-demographic, health and morbidity, household characteristics etc. It also included data on blood glucose, blood pressure and the height and weight of the sample mentioned above. This study provides data on 748,281 adults comprising 648,716 women and 99,565 men aged 15–49 years. The exclusion included 32,428 pregnant women and 22,815 missing and flagged data on various variables. Informed consent for participation in the survey and biomarker measurements and tests is taken from eligible women and men. During the survey, field staff read the informed consent statement precisely as written in the questionnaire. This statement explains the purpose of the study. It assures the respondents that their participation in the survey is entirely voluntary. They can refuse to answer any questions or stop the interview at any point. Health investigators collected biomarker measurements and tests from eligible men and women aged 15–49^27^. The data set is available in the public domain and same can be downloaded from the http://www.dhsprogram.com/. The NFHS data sets are anonymised, and hence participants can not be identified by the researchers.

## Description of variables

### Outcome variable

In our multivariate logistic regressions, the dependent variable is a binary variable that shows whether a person has diabetes. A person is considered diabetic if his/her non-fasting blood glucose level is greater than or equal to 200 mg/dl, or fasting glucose level is greater than or equal to 126 mg/dl. Those who reported being taking medicines for the treatment of diabetes are also considered diabetic.

### Independent variables

For the analysis in the first model, different socio-economic and demographic variables, namely the place of residence, religion, social category, wealth index, education in terms of years of schooling, age and marital status, have been treated as independent variables. Place of residence includes whether the households are in rural or urban areas. Social categories include general, scheduled castes (SCs), scheduled tribes (STs), other backward classes (OBCs), and do not know (includes missing data on the same along with those who reported not knowing their social category). The wealth index variable categorises households based on whether they belong to the poorest, poorer, middle, richer, or richest section of the society. For computation of the wealth index, households were given scores based on their performances as per several indicators covered under various dimensions. These dimensions included possession of durable consumer goods, materials used for household construction, water and sanitation facilities. For the derivation of scores, principal component analysis was used. Then, household scores were assigned to each respective household member, ranking each member in the household by their score and dividing the distribution into five equal categories (poorest, poorer, middle, richer and richest), each with 20 per cent of the population. The 1st quintile includes the twenty percent most impoverished individuals, the 2nd quintile contains twenty per cent of the second poorest individuals, and so on.

Further, religion includes Hindus, Muslims, Christians, Sikhs, and others (Buddhism, Jainism, Zoroastrianism and others). Based on educational qualification, individuals have been categorised as whether they have no schooling and have completed more than 12 years of education or less than equal. Based on age, individuals have been classified as to whether they fall into (15–19) years, (20–29) years, (30–39) years, or (40–49) years category. Furthermore, we have also included marital status as an independent variable, i.e. currently married, never married (includes married but never stayed with their spouses) and others (widowed, divorced, separated, and deserted).

The second model has been adjusted to all the socio-economic and demographic variables and risk factors responsible for the occurrence or stimulation of diabetes. These risk factors are hypertension, body mass index (BMI), tobacco use, alcohol use, vegetarian status, and frequency of watching television (TV). The hypertension variable is categorised as to whether the individual is suffering from hypertension or not. Those women and men whose systolic blood pressure is greater than or equal to 140 mmHg or diastolic blood pressure is greater than equal to 90 mmHg are considered hypertensive. Further, women and men who reported taking medicines to lower their blood pressure have also considered being hypertensive. BMI is categorized as too thin (BMI <  = 18.50), normal (18.50 < BMI <  = 24.99), overweight (25.00 < BMI <  = 29.99) and obese (BMI >  = 30). Further, tobacco and alcohol use has been categorised as to whether individuals consume or smoke these products or not. This study categorised women and men as vegetarians, non-vegetarians and vegans based on the frequency of some specific food items. Vegetarians have been defined as those who reported consuming milk or curd, green vegetables, fruits, pulses, or beans daily, weekly, or occasionally but no egg, fish, chicken, or meat. Non-vegetarians are those who reported consuming fruits, green vegetables, pulses or beans and animal products (including milk or curd, egg, fish, chicken, or meat) either daily, weekly, or occasionally. Vegans are those who reported not consuming animal products. Further, a sedentary lifestyle has been captured by the frequency of watching television which has categorised adults into two categories—those who reported to watch television not very frequently and those who reported watching television almost every day.

## Methods

To examine the gender-differential in the burden of diabetes, we have first calculated the prevalence of diabetes across different socio-economic and demographic variables, dietary habits, lifestyle and biological conditions. We have estimated the prevalence of diabetes among women and men (excluding pregnant women, flagged and missing values) surveyed in NFHS-4, consuming different types of foods with different frequencies.

$$\mathrm{Prevalence\, rate }=\frac{\mathrm{no}.\,\mathrm{ of\, women\, or \,men \,suffering\, from \,diabetes}}{\mathrm{total \,women\, or\, men\, population}}*100$$

We have applied separate regression analyses for women, men, and at all India level to capture gender-differential in the burden of diabetes. We have run two regression models for each category. The first model has been adjusted to socio-economic and demographic variables only, and the second model has been adjusted to all the socio-economic and demographic variables and risk factors for diabetes.

The multivariate logistic regression model I is noted as:

1 $$\frac{p}{1-p}={B}_{0}+{B}_{1}{X}_{1}+{B}_{2}{X}_{2}+{B}_{3}{X}_{3}+{B}_{4}{X}_{4}+{B}_{5}{X}_{5}+{B}_{6}{X}_{6}+{B}_{7}{X}_{7}+{u}_{i}$$

Where p is the probability of suffering from diabetes and 1-p is the probability of not suffering from diabetes. ${X}_{1}$ denotes the place of residence, ${X}_{2}$ denotes age group, ${X}_{3}$ represents the social category, ${X}_{4}$ represents religion, ${X}_{5}$ represents years of schooling completed, ${X}_{6}$ represents wealth index, ${X}_{7}$ represents marital status, ${u}_{i}$ is random error term and ${B}_{1}$, ${B}_{2}$,…,${B}_{7}$ are parameters to be estimated.

Further, the second multivariate logistic regression model is noted as:

2 $$\frac{p}{1-p}={B}_{0}+{{B}_{1}{X}_{1}+{B}_{2}{X}_{2}+{B}_{3}{X}_{3}+{B}_{4}{X}_{4}+{B}_{5}{X}_{5}+{B}_{6}{X}_{6}+{B}_{7}{X}_{7}+A}_{1}{y}_{1}+{A}_{2}{y}_{2}+{A}_{3}{y}_{3}+{A}_{4}{y}_{4}+{ A}_{5}{y}_{5}+{A}_{6}{y}_{6} +{u}_{i}$$

where ${y}_{1}$ denotes hypertension, ${y}_{2}$ denotes BMI, ${y}_{3}$ denotes the use of tobacco, ${y}_{4}$ represents the use of alcohol, ${y}_{5}$ represents vegetarian status, ${y}_{6}$ represents the frequency of watching television (TV) and ${A}_{1}$, ${A}_{2}$,…,${A}_{6}$ are parameters to be estimated.

The association between different groups and the probability of suffering from diabetes in both models is done through odds ratios.

### Ethical considerations

No separate ethical approval was needed as the study is based on secondary data obtained from the fourth round of the National Family Health Survey (NFHS-4). NFHS-4 data was collected following all the ethical clearance guidelines and norms. Its protocol was approved by the Institutional Review Board of the International Institute for Population Sciences (IIPS) and ICF International.

## Results and discussion

Table 1 presents the sample distribution and prevalence of diabetes among women and men between the age group 15–49, across different socio-economic and demographic variables. It shows that the overall prevalence of diabetes among women, men, and all India levels is 2.35%, 2.63%, and 2.39%, respectively, which shows that diabetes is higher among men than women in the country. The prevalence of diabetes is higher among urban adults (3.48%) than their rural counterparts. Among different gender, urban women (3.53%), and rural men (2.30%) are more affected than their respective counterparts. Among age groups, diabetes is primarily prevalent among the higher age group adults (6.28%), with a higher prevalence among men (6.91%) than women (6.18%). Among different religious groups, Christian adults (3.87%) are more affected than other groups. Among different gender in the same religious group, women (3.92%) have more diabetes than men (3.54%). Results of different social categories show that adults belonging to the do not know category (3.29%) are more affected by diabetes, followed by the general (2.78%) and OBCs (2.39%). Among different genders in the same social categories, women belonging to the do not know category (3.32%) are most affected, followed by the general category (2.73%). In contrast, for men, we saw the reverse trend. Among all wealth quintiles, adults belonging to the richest households (3.87%), especially men (4.04%), show a higher prevalence of diabetes than others. Furthermore, the prevalence of diabetes is higher among those adults who have no schooling (2.50%) than their other counterparts. In the same category, the gender-wise result shows that women with no schooling (2.51%) are more affected by diabetes whereas, in the case of men, it is higher among those who have schooling but less than 12 years (2.74%). Different marital statuses show that adults belonging to the other category (4.26%) are more affected by diabetes than their counterparts. Among different genders in the same category, the prevalence rate is higher among men (5.53%) than women (4.20%).

**Table 1:** Sample distribution and prevalence of diabetes among different socioeconomic and demographic groups in women and men of age group 15–49 years in National Family Health Survey, 2015–2016. All the values are weighted using sample survey weights from NFHS-4 and values in the parenthesis at all India level are unweighted.

Characteristics	Women participants (%)	Diabetes cases (%)	Prevalence rate of diabetes (Rate per 100)	Men participants (%)	Diabetes cases (%)	Prevalence rate of diabetes (Rate per 100)	Total participants (%)	Diabetes cases (%)	Prevalence rate of diabetes (Rate per 100)
All India	6,48,716	15,233 (12,530)	2.35	99,565	26,14 (2,318)	2.63	7,48,281	17,847 (14,848)	2.39
**Residence type**									
Urban	222,062 (34.23)	7833 (51.42)	3.53	37,113 (37.28)	1180 (45.12)	3.18	259,176 (34.64)	9013 (50.50)	3.48
Rural	426,654 (65.77)	7399 (48.58)	1.73	62,452 (62.72)	1435 (54.88)	2.30	489,105 (65.36)	8834 (49.50)	1.81
**Age group**									
15–19	113,725 (17.52)	365 (2.40)	0.32	18,076 (18.15)	54 (2.07)	0.30	131,801 (17.61)	419 (2.35)	0.32
20–29	208,278 (32.11)	1611 (10.57)	0.77	31,357 (31.49)	240 (9.17)	0.76	239,635 (32.03)	1851 (10.37)	0.77
30–39	178,572 (27.53)	4101 (26.92)	2.30	27,431 (27.56)	751 (28.72)	2.74	206,002 (27.53)	4852 (27.18)	2.36
40–49	148,141 (22.84)	9156 (60.11)	6.18	22,702 (22.80)	1570 (60.04)	6.91	170,843 (22.83)	10,725 (60.10)	6.28
**Religion**									
Hindu	524,249 (80.81)	11,492 (75.44)	2.19	81,458 (81.81)	2111 (80.75)	2.59	605,707 (80.95)	13,603 (76.22)	2.25
Muslim	87,523 (13.49)	2640 (17.33)	3.02	12,880 (12.94)	344 (13.14)	2.67	100,403 (13.42)	2984 (16.72)	2.97
Christian	15,418 (2.38)	604 (3.97)	3.92	2196 (2.21)	78 (2.97)	3.54	17,613 (2.35)	682 (3.82)	3.87
Sikh	11,031 (1.70)	245 (1.61)	2.22	1597 (1.60)	38 (1.45)	2.38	12,628 (1.69)	283 (1.59)	2.24
Others	10,495 (1.62)	251 (1.65)	2.39	1434 (1.44)	44 (1.69)	3.08	11,929 (1.59)	295 (1.65)	2.47
**Social category**									
General	145,429 (22.42)	3967 (26.04)	2.73	22,738 (22.84)	711 (27.18)	3.12	168,167 (22.47)	4677 (26.21)	2.78
SCs	132,428 (20.41)	2773 (18.21)	2.09	19,867 (19.95)	471 (18.04)	2.37	152,295 (20.35)	3244 (18.18)	2.13
STs	59,721 (9.21)	876 (5.75)	1.47	8889 (8.93)	153 (5.85)	1.72	68,610 (9.17)	1029 (5.77)	1.50
OBCs	282,410 (43.53)	6663 (43.74)	2.36	43,710 (43.90)	1144 (43.74)	2.62	326,120 (43.58)	7807 (43.74)	2.39
Don’t know	28,728 (4.43)	954 (6.26)	3.32	4362 (4.38)	136 (5.19)	3.11	33,090 (4.42)	1089 (6.10)	3.29
**Wealth index**									
Poorest	114,492 (17.65)	1212 (7.96)	1.06	14,888 (14.95)	199 (7.62)	1.34	129,380 (17.29)	1411 (7.91)	1.09
Poorer	127,723 (19.69)	1779 (11.68)	1.39	18,986 (19.07)	319 (12.18)	1.68	146,709 (19.61)	2098 (11.76)	1.43
Middle	134,068 (20.67)	2622 (17.21)	1.96	21,283 (21.38)	514 (19.67)	2.42	155,351 (20.76)	3136 (17.57)	2.02
Richer	137,566 (21.21)	4431 (29.09)	3.22	21,934 (22.03)	675 (25.81)	3.08	159,500 (21.31)	5106 (28.61)	3.20
Richest	134,868 (20.78)	5188 (34.06)	3.85	22,473 (22.57)	908 (34.72)	4.04	157,341 (21.03)	6096 (34.16)	3.87
**Education level**									
No schooling	180,832 (27.88)	4547 (29.85)	2.51	12,032 (12.08)	271 (10.38)	2.26	192,864 (25.77)	4819 (27.00)	2.50
< = 12 years	331,286 (51.07)	7994 (52.48)	2.41	58,319 (58.57)	1597 (61.09)	2.74	389,606 (52.07)	9591 (53.74)	2.46
> 12 years	136,598 (21.06)	2691 (17.67)	1.97	29,214 (29.35)	746 (28.54)	2.55	165,811 (22.16)	3437 (19.26)	2.07
**Marital status**									
Never married	152,644 (23.53)	743 (4.88)	0.49	37,721 (37.89)	250 (9.58)	0.66	190,366 (25.44)	994 (5.57)	0.52
Currently married	467,733 (72.10)	13,299 (87.31)	2.84	60,583 (60.84)	2294 (87.75)	3.79	528,316 (70.60)	15,593 (87.37)	2.95
Others	28,339 (4.37)	1190 (7.81)	4.20	1260 (1.27)	70 (2.67)	5.53	29,599 (3.96)	1260 (7.06)	4.26

Table 2 shows the prevalence of diabetes among women and men having a different frequency of intake of several food items. Adults who reported consuming milk or curd daily (2.79%) have more diabetes compared to others, with a relatively higher prevalence among men (2.82%). Further, diabetes is most prevalent among adults who reported never consuming pulses or beans. Its prevalence is higher among women (2.66%) who never consume the same food item in different genders. Still, among men, the prevalence of diabetes is higher among those who reported consuming these food items occasionally (2.85%). It is also observed that women and men who reported consuming green vegetables and fruits daily are most affected by diabetes (2.44%, 3.48%; 2.67%, 3.51%) with differences in magnitudes. Diabetes is most prevalent among egg consumers, especially women (3.66%) who reported consuming it daily.

**Table 2:** Sample distribution and prevalence of diabetes among women and men of age group 15–49 years as per frequency of intake of food items in National Family Health Survey, 2015–2016. All the values are weighted using sample survey weights from NFHS-4.

Frequency of intake	Women participants (%)	Diabetes cases and its prevalence among different categories (%)	Men participants (%)	Diabetes cases and its prevalence among different categories (%)	Total participants (%)	Diabetes cases and its prevalence among different categories (%)
**Milk or curd**						
Never	47,927 (7.39)	950 (1.98)	5080 (5.10)	111 (2.19)	53,007 (7.08)	1062 (2.00)
Daily	289,594 (44.64)	8054 (2.78)	46,056 (46.26)	1300 (2.82)	335,650 (44.86)	9354 (2.79)
Weekly	149,051 (22.98)	3327 (2.23)	28,469 (28.59)	732 (2.57)	177,520 (23.72)	4059 (2.29)
Occasionally	162,145 (24.99)	2902 (1.79)	19,960 (20.05)	471 (2.36)	182,104 (24.34)	3373 (1.85)
**Pulses or beans**						
Never	3672 (0.57)	98 (2.66)	418 (0.42)	10 (2.29)	4089 (0.55)	108 (2.62)
Daily	290,265 (44.74)	6864 (2.36)	46,376 (46.58)	1233 (2.66)	336,642 (44.99)	8097 (2.41)
Weekly	292,618 (45.11)	7003 (2.39)	43,836 (44.03)	1117 (2.55)	336,455 (44.96)	8120 (2.41)
Occasionally	62,161 (9.58)	1268 (2.04)	8935 (8.97)	254 (2.85)	71,095 (9.5)	1522 (2.14)
**Green leafy vegetables**						
Never	2559 (0.39)	61 (2.40)	461 (0.46)	8 (1.66)	3020 (0.4)	69 (2.28)
Daily	306,447 (47.24)	7466 (2.44)	46,569 (46.77)	1244 (2.67)	353,017 (47.18)	8710 (2.47)
Weekly	247,979 (38.23)	5832 (2.35)	41,306 (41.49)	1095 (2.65)	289,286 (38.66)	6927 (2.39)
Occasionally	91,730 (14.14)	1873 (2.04)	11,229 (11.28)	268 (2.38)	102,959 (13.76)	2141 (2.08)
**Fruits**						
Never	16,747 (2.58)	440 (2.63)	1910 (1.92)	62 (3.23)	18,657 (2.49)	502 (2.69)
Daily	77,193 (11.9)	2689 (3.48)	10,787 (10.83)	379 (3.51)	87,980 (11.76)	3068 (3.49)
Weekly	214,842 (33.12)	5488 (2.55)	39,202 (39.37)	1123 (2.86)	254,044 (33.95)	6611 (2.60)
Occasionally	339,934 (52.4)	6616 (1.95)	47,666 (47.88)	1051 (2.2)	387,600 (51.80)	7667 (1.98)
**Eggs**						
Never	190,980 (29.44)	3602 (1.89)	19,730 (19.82)	453 (2.29)	210,709 (28.16)	4055 (1.92)
Daily	25,109 (3.87)	918 (3.66)	4818 (4.84)	145 (3.00)	29,927 (4.00)	1063 (3.55)
Weekly	242,158 (37.33)	6528 (2.7)	44,202 (44.39)	1330 (3.01)	286,360 (38.27)	7858 (2.74)
Occasionally	190,469 (29.36)	4184 (2.2)	30,816 (30.95)	687 (2.23)	221,285 (29.57)	4871 (2.2)
**Fish**						
Never	228,824 (35.27)	4110 (1.8)	27,341 (27.46)	549 (2.01)	256,165 (34.23)	4659 (1.82)
Daily	36,392 (5.61)	1391 (3.82)	4742 (4.76)	195 (4.12)	41,134 (5.50)	1586 (3.86)
Weekly	184,212 (28.4)	5266 (2.86)	33,391 (33.54)	1046 (3.13)	217,604 (29.08)	6313 (2.90)
Occasionally	199,287 (30.72)	4466 (2.24)	34,091 (34.24)	824 (2.42)	233,378 (31.19)	5289 (2.27)
**Chicken or meat**						
Never	207,550 (31.99)	3774 (1.82)	23,116 (23.22)	494 (2.14)	230,667 (30.83)	4268 (1.85)
Daily	6804 (1.05)	241 (3.54)	1656 (1.66)	36 (2.17)	8461 (1.13)	277 (3.27)
Weekly	202,783 (31.26)	5943 (2.93)	38,403 (38.57)	1276 (3.32)	241,186 (32.23)	7219 (2.99)
Occasionally	231,579 (35.70)	5275 (2.28)	36,389 (36.55)	808 (2.22)	267,968 (35.81)	6083 (2.27)

In comparison, fish consumers' results show that men consuming it daily (4.12%) are more affected than women (3.82%). Also, adults consuming chicken or meat daily are most affected as compared to others. Among different gender, women who consume chicken or meat daily (3.54%) are most affected by diabetes, and in the case of men, diabetes is high among those who consume the same weekly (3.32%).

Results in Table 3 reveal that both in rural and urban areas, the percentage of non-vegetarians is higher than vegetarians and vegans, which constituted 76.69% in urban and 73.75% in rural areas. Among different genders in urban and rural areas, non-vegetarians are higher among men (83.26%; 81.92%) than women (75.6%; 72.55%). While, among different age groups, this figure is higher among 20–29 years old (75.83%) with a higher percentage among men (83.81%) as compared to women (74.63%). Further, among different religious groups, the rate of non-vegetarians is higher among Christian adults. Among different genders, the percentage of non-vegetarians is higher among Christian women and Muslim men (98.69% and 99.12%), followed by Muslim women and Christian men (98.33% and 98.78%) and other categories (85.12% women and 87.32% men).

**Table 3:** Percentage distribution of different socioeconomic and demographic groups according to types of vegetarian diet consumption in women and men participants in National Family Health Survey, 2015–2016. All the values are weighted using sample survey weights from NFHS-4.

Characteristics	Women			Men			Total		
	Non-vegetarian (%)	Vegetarian (%)	Vegan (%)	Non-vegetarian (%)	Vegetarian (%)	Vegan (%)	Non-vegetarian (%)	Vegetarian (%)	Vegan (%)
**Place of residence**									
Urban	167,870 (75.60)	51,824 (23.34)	2369 (1.06)	30,899 (83.26)	6047 (16.29)	168 (0.45)	198,768 (76.69)	57,870 (22.33)	2537 (0.98)
Rural	309,555 (72.55)	110,445 (25.89)	6654 (1.56)	51,157 (81.92)	10,831 (17.34)	464 (0.74)	360,712 (73.75)	121,276 (24.80)	7118 (1.45)
**Age group**									
15–19	82,945 (72.94)	28,702 (25.24)	2077 (1.82)	14,814 (81.96)	3161 (17.49)	101 (0.56)	97,760 (74.17)	31,863 (24.18)	2178 (1.65)
20–29	155,443 (74.63)	50,192 (24.10)	2644 (1.27)	26,279 (83.81)	4899 (15.62)	178 (0.57)	181,722 (75.83)	55,091 (22.99)	2822 (1.18)
30–39	132,267 (74.07)	44,005 (24.64)	2300 (1.29)	22,583 (82.33)	4667 (17.01)	181 (0.66)	154,850 (75.17)	48,672 (23.63)	2481 (1.20)
40–49	106,769 (72.07)	39,370 (26.58)	2002 (1.35)	18,380 (80.96)	4150 (18.28)	172 (0.76)	125,149 (73.26)	43,520 (25.47)	2174 (1.27)
**Religion**									
Hindu	363,526 (69.34)	152,267 (29.05)	8456 (1.61)	64,857 (79.62)	16,006 (19.65)	594 (0.73)	428,383 (70.72)	168,273 (27.78)	9051 (1.50)
Muslim	86,067 (98.33)	1284 (1.47)	171 (0.20)	12,767 (99.12)	100 (0.77)	14 (0.11)	98,834 (98.44)	1384 (1.38)	185 (0.18)
Christian	15,216 (98.69)	188 (1.22)	14 (0.09)	2169 (98.78)	17 (0.78)	10 (0.44)	17,385 (98.71)	205 (1.16)	24 (0.13)
Sikh	3682 (33.38)	7096 (64.33)	253 (2.29)	1011 (63.28)	577 (36.13)	9 (0.59)	4693 (37.16)	7673 (60.76)	262 (2.08)
Others	8934 (85.12)	1433 (13.66)	128 (1.22)	1252 (87.32)	177 (12.35)	5 (0.33)	10,186 (85.39)	1610 (13.50)	133 (1.11)
**Social category**									
General	93,543 (64.32)	49,948 (34.35)	1939 (1.33)	16,809 (73.93)	5788 (25.45)	141 (0.62)	110,352 (65.62)	55,736 (33.14)	2079 (1.24)
SCs	108,334 (81.80)	22,243 (16.80)	1850 (1.40)	17,688 (89.03)	2073 (10.43)	106 (0.54)	126,022 (82.75)	24,316 (15.97)	1957 (1.28)
STs	49,918 (83.58)	8660 (14.51)	1143 (1.91)	7934 (89.25)	863 (9.71)	92 (1.04)	57,852 (84.32)	9523 (13.88)	1235 (1.80)
OBCs	199,363 (70.59)	79,124 (28.02)	3923 (1.39)	35,519 (81.26)	7918 (18.12)	272 (0.62)	234,882 (72.02)	87,043 (26.69)	4195 (1.29)
Don’t know	26,267 (91.44)	2293 (7.98)	167 (0.58)	4105 (94.12)	236 (5.41)	21 (0.47)	30,373 (91.79)	2529 (7.64)	188 (0.57)
**Wealth index**									
Poorest	91,168 (79.63)	21,142 (18.46)	2182 (1.91)	12,922 (86.80)	1798 (12.07)	168 (1.13)	104,090 (80.45)	22,940 (17.73)	2350 (1.82)
Poorer	98,185 (76.87)	27,479 (21.51)	2058 (1.62)	16,068 (84.63)	2768 (14.58)	151 (0.79)	114,253 (77.88)	30,247 (20.62)	2209 (1.51)
Middle	102,083 (76.14)	30,186 (22.52)	1799 (1.34)	18,022 (84.68)	3131 (14.71)	131 (0.61)	120,105 (77.31)	33,317 (21.45)	1930 (1.24)
Richer	102,182 (74.28)	33,766 (24.55)	1618 (1.18)	18,246 (83.18)	3603 (16.43)	85 (0.39)	120,428 (75.50)	37,369 (23.43)	1703 (1.07)
Richest	83,807 (62.14)	49,696 (36.85)	1365 (1.01)	16,798 (74.75)	5578 (24.82)	97 (0.43)	100,605 (63.94)	55,274 (35.13)	1462 (0.93)
**Education level**									
No schooling	138,042 (76.34)	39,955 (22.10)	2835 (1.56)	10,623 (88.29)	1301 (10.81)	108 (0.90)	148,666 (77.08)	41,256 (21.39)	2943 (1.53)
< = 12 years	247,647 (74.75)	78,850 (23.80)	4790 (1.45)	48,399 (82.99)	9537 (16.35)	383 (0.66)	296,046 (75.98)	88,387 (22.69)	5173 (1.33)
> 12 years	91,735 (67.16)	43,464 (31.82)	1398 (1.02)	23,034 (78.85)	6039 (20.67)	141 (0.48)	114,769 (69.22)	49,504 (29.85)	1539 (0.93)
**Marital status**									
Never married	110,273 (72.24)	39,797 (26.07)	2574 (1.69)	31,334 (83.07)	6198 (16.43)	189 (0.50)	141,608 (74.39)	45,995 (24.16)	2763 (1.45)
Currently married	345,174 (73.80)	116,542 (24.91)	6017 (1.29)	49,721 (82.07)	10,439 (17.23)	424 (0.70)	394,894 (74.75)	126,981 (24.03)	6441 (1.22)
Others	21,977 (77.55)	5930 (20.92)	432 (1.52)	1001 (79.41)	241 (19.08)	19 (1.51)	22,978 (77.63)	6170 (20.85)	451 (1.52)

Furthermore, different social categories show that the percentage of non-vegetarians is higher than vegetarians and vegans among each type. It is maximum among the do not know category (91.79%) followed by STs (84.32%) and SCs (82.75%) with a similar trend among both the genders with a higher share among men. Among different wealth quintiles, the percentage of non-vegetarians is higher than vegetarians and vegans. This figure is higher among adults falling in the poorest category (80.45%), with a higher percentage among men than women. Among different education levels, the percentage of non-vegetarians is higher among no schooling adults (77.08%) with a higher percentage of men. Among different marital status categories, the percentage of non-vegetarians in all categories (74.39% never married, 74.75% currently married and 77.63% Others) is higher than vegetarians and vegans. In the same marital status category, the percentage of non-vegetarians is higher among others (77.63%) than their counterparts. These figures are in the same line in both genders, with a higher magnitude in men than women.

Table 4 shows that diabetes is more prevalent among hypertensive and obese adults than other respective categories. Among different genders, the prevalence of diabetes is higher among obese men (11.46%) than obese women (9.19%). Further, tobacco and alcohol consumers (2.74 and 3.41%) are more affected by diabetes than their counterparts (2.34 and 2.33%). In the same line, men and women who consume alcohol or smoke or consume tobacco are more affected by diabetes (3.33% and 3.71%; 2.77% and 2.71%) than their counterparts. The analysis also showed that diabetes is more prevalent among non-vegetarians (2.58%) as compared to vegetarians (1.81%) and vegans (1.58%), with a higher prevalence among non-vegetarian men (2.71%) than women (2.56%). It is also clear from the results that those who watch television (TV) almost every day, especially men (3.03%), are more affected by diabetes than their counterparts.

**Table 4:** Sample distribution and prevalence of diabetes in different risk factors for diabetes among women and men in National Family Health Survey, 2015–2016. All the values are weighted using sample survey weights from National Family Health Survey-4 and values in the parenthesis at all India level are unweighted.

Characteristics	Women			Men			Total		
	Frequency (%)	Diabetes (%)	Prevalence of diabetes (rate per 100)	Frequency (%)	Diabetes (%)	Prevalence of diabetes (rate per 100)	Frequency (%)	Diabetes (%)	prevalence of diabetes (rate per 100)
All India	6,48,716	15,233 (12,530)	2.35	99,565	26,14 (2,318)	2.63	7,48,281	17,847 (14,848)	2.39
**Hypertension**									
No	569,109 (87.73)	9750 (64.01)	1.71	83,541 (83.91)	1543 (59.01)	1.85	652,650 (87.22)	11,293 (63.27)	1.73
Yes	79,607 (12.27)	5483 (35.99)	6.89	16,024 (16.09)	1071 (40.99)	6.69	95,631 (12.78)	6554 (36.73)	6.85
**Body mass index**									
Too thin	148,246 (22.85)	1006 (6.60)	0.68	20,089 (20.18)	181 (6.92)	0.90	168,335 (22.50)	1187 (6.65)	0.71
Normal	367,029 (56.58)	5990 (39.33)	1.63	60,666 (60.93)	1168 (44.66)	1.92	427,695 (57.15)	7158 (40.11)	1.67
Overweight	100,347 (15.47)	5194 (34.10)	5.18	15,790 (15.86)	920 (35.18)	5.82	116,137 (15.52)	6114 (34.26)	5.26
Obese	33,095 (5.10)	3042 (19.97)	9.19	3020 (3.03)	346 (13.24)	11.46	36,115 (4.83)	3388 (18.98)	9.38
**Consume/smoke tobacco**									
Yes	45,278 (6.98)	1226 (8.05)	2.71	44,778 (44.97)	1242 (47.50)	2.77	90,055 (12.03)	2468 (13.83)	2.74
No	603,438 (93.02)	14,007 (91.95)	2.32	54,787 (55.03)	1372 (52.50)	2.50	658,226 (87.97)	15,379 (86.17)	2.34
**Drinks alcohol**									
No	640,590 (98.75)	14,932 (98.02)	2.33	70,237 (70.54)	1639 (62.70)	2.33	710,826 (94.99)	16,570 (92.85)	2.33
Yes	8126 (1.25)	301 (1.98)	3.71	29,328 (29.46)	975 (37.30)	3.33	37,455 (5.01)	1277 (7.15)	3.41
**Types of vegetarians**									
Non-vegetarian	477,424 (73.60)	12,227 (80.27)	2.56	82,056 (82.42)	2228 (85.20)	2.71	559,480 (74.77)	14,454 (80.98)	2.58
Vegetarian	162,269 (25.01)	2863 (18.79)	1.76	16,877 (16.95)	378 (14.45)	2.24	179,146 (23.94)	3240 (18.16)	1.81
Vegan	9023 (1.39)	144 (0.94)	1.59	632 (0.63)	9 (0.35)	1.44	9654 (1.29)	153 (0.86)	1.58
**Frequency of watching TV**									
Not frequent	251,430 (38.76)	4207 (27.62)	1.67	37,714 (37.88)	739 (28.27)	1.96	289,143 (38.64)	4946 (27.71)	1.71
Almost every day	397,286 (61.24)	11,026 (72.38)	2.78	61,851 (62.12)	1875 (71.74)	3.03	459,138 (61.36)	12,901 (72.29)	2.81

In Table 5, the multivariate regression analysis (model I) shows that adults residing in rural areas are significantly less likely to suffer from diabetes than their urban counterparts. It is also clear that with an increase in age, the likelihood of suffering from diabetes increases among both women and men. Among different religious groups, the likelihood of being affected by diabetes is significantly more among Muslim adults (OR:1.45). The gender-wise analysis shows that Muslim women (OR: 1.52) and Christian men are at greater risk, but the second results are not significant. Further, the study shows that women from scheduled caste (OR: 1.09) are more likely to be diabetic, whereas results are not significant for men. Among different wealth quintiles, women and men from the richest section (OR: 2.62 and OR: 2.25) are significantly more likely to suffer from diabetes than their respective counterparts.

**Table 5:** Multivariate Logistic regression table showing the association between diabetes and different socioeconomic and demographic variables and risk factors.

Characteristics	Women				Men				Total			
	Number of observations = 6,48,716				Number of observations = 99,565				Number of observations = 7,48,281			
	Model I^1^		Model II^2^		Model I		Model II		Model I		Model II	
	Odds Ratio (CI)	P > z	Odds Ratio (CI)	P > z	Odds Ratio (CI)	P > z	Odds Ratio (CI)	P > z	Odds Ratio (CI)	P > z	Odds Ratio (CI)	P > z
**Place of residence**												
Urban	1		1		1		1		1		1	
Rural	0.77 (0.74–0.80)	0.000	0.86 (0.82–0.89)	0.000	0.96 (0.87–1.06)	0.414*	1.01 (0.92–1.12)	0.769*	0.8 (0.77–0.83)	0.000	0.88 (0.85–0.92)	0.000
**Age group**												
15–29	1		1		1		1		1		1	
20–29	1.76 (1.53–2.02)	0.000	1.55 (1.35–1.78)	0.000	1.73 (1.32–2.26)	0.000	1.56 (1.19–2.05)	0.001	1.8 (1.59–2.03)	0.000	1.56 (1.38–1.76)	0.000
30–39	5.13 (4.46–5.91)	0.000	3.58 (3.10–4.12)	0.000	4.72 (3.55–6.28)	0.000	3.77 (2.82–5.04)	0.000	5.31 (4.69–6.02)	0.000	3.67 (3.23–4.16)	0.000
40–49	14.35 (12.48–16.5)	0.000	8.62 (7.48–9.93)	0.000	11.41 (8.58–15.18)	0.000	8.58 (6.40–11.48)	0.000	14.6 (12.9–16.53)	0.000	8.81 (7.76–9.99)	0.000
**Religion**												
Hindu	1		1		1		1		1		1	
Muslim	1.52 (1.44–1.6)	0.000	1.22 (1.16–1.29)	0.000	1.06 (0.92–1.21)	0.435*	1.04 (0.90–1.19)	0.623*	1.45 (1.38–1.52)	0.000	1.21 (1.15–1.27)	0.000
Christian	1.16 (1.06–1.26)	0.001	1.03 (0.94–1.12)	0.543*	1.12 (0.93–1.35)	0.242*	1.06 (0.88–1.28)	0.524*	1.13 (1.05–1.22)	0.002	1.02 (0.94–1.10)	0.660*
Sikh	0.80 (0.71–0.89)	0.000	0.78 (0.69–0.87)	0.000	0.79 (0.59–1.06)	0.120*	0.69 (0.51–0.92)	0.013	0.79 (0.71–0.88)	0.000	0.76 (0.68–0.85)	0.000
Others	0.92 (0.81–1.05)	0.236*	0.8 (0.70–0.91)	0.001	0.72 (0.53–0.98)	0.036	0.63 (0.46–0.86)	0.003	0.89 (0.78–1.00)	0.046	0.76 (0.67–0.86)	0.000
**Social category**												
General	1		1		1		1		1		1	
SC	1.09 (1.03–1.16)	0.004	1.04 (0.98–1.10)	0.202*	0.93 (0.81–1.07)	0.335*	0.92 (0.80–1.06)	0.233*	1.07 (1.02–1.13)	0.011	1.03 (0.97–1.09)	0.356*
ST	0.74 (0.68–0.8)	0.000	0.72 (0.67–0.78)	0.000	0.88 (0.75–1.03)	0.117*	0.85 (0.72–1.01)	0.058*	0.77 (0.72–0.82)	0.000	0.74 (0.69–0.8)	0.000
OBC	0.99 (0.95–1.04)	0.819*	1.00 (0.95–1.05)	0.991*	0.90 (0.8–1.00)	0.051	0.90 (0.81–1.01)	0.072*	0.98 (0.94–1.03)	0.486*	0.99 (0.95–1.03)	0.614*
Don’t know	1.07 (0.98–1.16)	0.137*	0.97 (0.89–1.06)	0.492*	0.97 (0.79–1.19)	0.788*	0.95 (0.78–1.17)	0.637*	1.05 (0.97–1.14)	0.240*	0.97 (0.89–1.05)	0.399*
**Wealth index**												
Poorest	1		1		1		1		1		1	
Poorer	1.21 (1.12–1.30)	0.000	1.09 (1.01–1.18)	0.031	1.16 (0.97–1.38)	0.099*	1.03 (0.86–1.23)	0.757*	1.2 (1.12–1.29)	0.000	1.09 (1.01–1.17)	0.023
Middle	1.58 (1.46–1.70)	0.000	1.27 (1.17–1.37)	0.000	1.46 (1.23–1.74)	0.000	1.13 (0.94–1.35)	0.203*	1.56 (1.46–1.67)	0.000	1.25 (1.17–1.35)	0.000
Richer	2.25 (2.09–2.43)	0.000	1.64 (1.51–1.78)	0.000	1.91 (1.60–2.27)	0.000	1.31 (1.09–1.59)	0.005	2.19 (2.05–2.35)	0.000	1.59 (1.48–1.72)	0.000
Richest	2.62 (2.42–2.83)	0.000	1.83 (1.67–2.00)	0.000	2.25 (1.87–2.71)	0.000	1.44 (1.18–1.77)	0.000	2.54 (2.36–2.73)	0.000	1.77 (1.63–1.92)	0.000
**Education level**												
No schooling	1		1		1		1		1		1	
< = 12 years	1.26 (1.21–1.32)	0.000	1.15 (1.10–1.20)	0.000	1.48 (1.28–1.72)	0.000	1.40 (1.21–1.63)	0.000	1.30 (1.25–1.36)	0.000	1.19 (1.14–1.24)	0.000
> 12 years	1.02 (0.95–1.09)	0.587*	0.95 (0.89–1.02)	0.140*	1.61 (1.36–1.91)	0.000	1.45 (1.22–1.72)	0.000	1.13 (1.07–1.20)	0.000	1.04 (0.98–1.11)	0.151*
**Marital status**												
Never married	1		1		1		1		1		1	
Currently married	1.40 (1.26–1.54)	0.000	1.24 (1.12–1.37)	0.000	1.33 (1.13–1.58)	0.001	1.23 (1.04–1.46)	0.017	1.32 (1.22–1.44)	0.000	1.20 (1.11–1.31)	0.000
Others	1.57 (1.40–1.77)	0.000	1.41 (1.25–1.59)	0.000	1.16 (0.80–1.69)	0.434*	1.18 (0.81–1.72)	0.388*	1.46 (1.32–1.63)	0.000	1.35 (1.21–1.5)	0.000
**Hypertension**												
No	–	–	1		–	–	1		–	–	1	
Yes	–	–	1.96 (1.88–2.04)	0.000	–	–	1.76 (1.61–1.93)	0.000	–	–	1.94 (1.87–2.01)	0.000
**Body mass index**												
Too thin	–	–	1		–	–	1		–	–	1	
Normal	–	–	1.41 (1.31–1.52)	0.000	–	–	1.19 (1.01–1.39)	0.036	–	–	1.38 (1.29–1.48)	0.000
Overweight	–	–	2.75 (2.54–2.97)	0.000	–	–	2.06 (1.73–2.45)	0.000	–	–	2.64 (2.46–2.84)	0.000
Obese	–	–	4.25 (3.90–4.63)	0.000	–	–	3.77 (3.06–4.65)	0.000	–	–	4.17 (3.86–4.51)	0.000
**Consume/smoke tobacco**												
Yes	–	–	1		–	–	1		–	–	1	
No	–	–	0.97 (0.92–1.03)	0.364*	–	–	1.12 (1.02–1.23)	0.017	–	–	0.98 (0.93–1.02)	0.303*
**Drinks alcohol**												
No	–	–	1		–	–	1		–	–	1	
Yes	–	–	0.99 (0.87–1.12)	0.883*	–	–	1.05 (0.96–1.16)	0.294*	–	–	1.11 (1.04–1.19)	0.002
**Types of vegetarian**												
Non–vegetarian	–	–	1		–	–	1		–	–	1	
vegetarian	–	–	0.66 (0.63–0.70)	0.000	–	–	0.76 (0.67–0.86)	0.000	–	–	0.67 (0.64–0.7)	0.000
vegan	–	–	0.89 (0.75–1.05)	0.171*	–	–	0.84 (0.46–1.54)	0.566*	–	–	0.89 (0.76–1.04)	0.138*
**Frequency of watching TV**												
Not very frequent	–	–	1		–	–	1		–	–	1	
Almost every day	–	–	1.08 (1.03–1.13)	0.001	–	–	1.21 (1.09–1.34)	0.000	–	–	1.10 (1.06–1.15)	0.000

Model I is adjusted to socioeconomic and demographic variables.

Model II is adjusted to all the risk factors along with socioeconomic and demographic variables.

*Not significant at 95% confidence interval.

Note: Values in parenthesis are confidence intervals.

The gender-wise analysis of different education levels shows that women with schooling less than or equal to 12 years (OR: 1.26) are significantly more at risk, but men with schooling for more than 12 years (1.61) are at greater risk. Analysis of different marital statuses shows that men in the ‘currently married’ (OR:1.33) category are at greater risk of diabetes than others. At the same time, in the case of women, the results are non-significant.

Model II, adjusted to socio-economic and demographic and risk factors, also presents almost the same picture in the case of women, men, and all India levels across different groups. Overall analysis shows that hypertensive and obese adults (OR: 1.94 and 4.17) are significantly most likely to get diabetes. The same is the pattern in women and men, i.e. hypertensive and obese women (OR: 1.96 and OR: 4.25) and men (OR: 1.76 and OR: 3.77) are at a greater risk. Further, it is also observed that adults consuming alcohol (OR: 1.11) and smoking tobacco are more likely to suffer from diabetes than those who do not drink alcohol and smoke tobacco, respectively. Further, vegetarian women (OR: 0.66) and men (OR: 0.76) are at lower risks than non-vegetarians. Model II also shows that the likelihood of suffering from diabetes is higher among those women and men who watch television almost every day (OR: 1.08 and OR: 1.21) than their respective counterparts.

Findings of our study show that the overall prevalence of diabetes is higher among men than women, which can be explained by adverse effects of dietary and lifestyle habits and physiological factors like increasing prevalence of hypertension and obesity among men. Our study also observed that urban adults are most likely to suffer from diabetes than rural adults. The reason could be a changed lifestyle in terms of eating habits and sedentary occupation in urban adults. Cities and towns are overwhelmed by the fast-food culture, a significant stimulus of diabetes as these foods are rich in calories and fat^28^. On the other hand, ‘sedentarism’ is also a considerable driver of diabetes. Over the year, people have shifted to jobs that demand less physical activity^29^. A study observed that the prevalence of diabetes was three times higher among individuals engaged in light physical work than those involved in heavy physical work^30^.

Further, our study indicates that the prevalence of diabetes is higher among urban women than urban men. It could be because of occupational stress, increasing carrier orientation, increasing domestic responsibilities because of nuclear families and other psychosocial factors. Studies have also shown that more household responsibilities and unpaid household work among women may cause feelings of conflict, contributing to high-stress level diabetes^31,32^.

We also found that the prevalence and likelihood of suffering from diabetes increase with the increase in age. The association could be that with an increase in age, there is an increase in insulin resistance, and pancreatic islet function gets compromised^33^. A study by the Indian Council of Medical Research (ICMR) has also shown that the prevalence of diabetes increases with an increase in age until 60 years, and after that, it falls^34^. Further, the lower prevalence of diabetes among women in higher age groups than men could be that in a country like India, women, even at a higher age, get associated with household and family chores, which keeps them physically active. In comparison, most men in rural areas in this age group prefer not to get associated with household chores, which might contribute to the higher prevalence of diabetes and other factors.

Christian and Muslim adults are most affected by diabetes, and it could be because of their lifestyle and eating habits. This result is consistent with few other studies showing a higher prevalence of diabetes among Christian adults in India^35^. It is also found in the literature that most Muslims consume foods rich in carbohydrates and sugar. Their calorie intake is also very high because of the frequent consumption of non-veg food^36^. Further, the sedentary life of most Muslim women makes them more vulnerable as compared to others. The finding that Christians have more diabetes is also verified by calculating the prevalence of risk factors like obesity, overweight, and physical inactivity among different religious groups. The results show a higher prevalence among Christians following Sikh among all religious groups in the country.

Our study also indicated that widowed, divorced, separated and deserted adults are most affected than their counterparts. This observation may be explained by the adverse effects of the burden of living and fulfilling the requirements of family members alone, which cause stress and sleep deprivation and intensifies diabetes.

With the growth of the economy, in terms of gross domestic product and per capita income over the years, the access to calorie-rich food among wealthy people has increased, which could be a reason for higher prevalence and more likelihood of suffering from diabetes among the richest section of India^37,38^. The justification for higher prevalence among the richest men than women could be that the richest men are mainly in the business and services sector, which requires less physical activity and a different diet, including fast foods.

It is also noticed that adults who consumed non-veg food daily, weekly, or occasionally are more likely to suffer from diabetes. One reason for this association could be the high level of cholesterol and saturated fat in the yolk and the more use of oil for cooking fish and chicken^39^. The higher prevalence among adults who reported never or occasionally consume pulses or beans could be because pulse consumption reduces postprandial blood glucose, and regular and long-term consumption helps improve glycemic control^40^. We believe that the association between the higher level of diabetes and daily consumption of green vegetables, milk or curd and fruits might not be causal. There could be other factors contributing to this association. It might be the case that some of these adults are taking other food items, some are not physically active and some are practicing both which is contributing to their diabetes.

Further, it is also observed that adults having hypertension and obesity are most likely to suffer from diabetes. Our finding is also supported by another study showing a causal association between blood pressure and diabetes. If people try or make efforts to lower blood pressure, it can reduce the incidence of diabetes^41^. In obese individuals, the amount of non-esterified fatty acids, glycerol, hormones, cytokines, pro-inflammatory markers, and other substances involved in the development of insulin resistance increases^42^. Further, obese men are more affected than obese women. This finding is in line with the results of other studies^6^. One possible explanation for this difference is that there are differences in fat distribution among men and women. Among men, it is stored in their liver and around the waist, whereas women tend to store it on their thighs and hips. The excess fat accumulation in organs like the liver and muscles affect the body’s ability to regulate sugar level. Therefore, men can develop diabetes with lesser weight gain than women^6,43^.

Furthermore, our study found that the prevalence of diabetes is higher among alcohol consumers than among non-alcohol consumers. Findings from other studies have also shown that alcohol consumption is a stimulus for diabetes. It is found that there is no relationship between smoking and diabetes. Alcohol consumption may be a significant independent factor for stimulating diabetes as too much alcohol consumption causes chronic inflammation of the pancreas, affecting its ability to secrete insulin^41,44^.

Like other studies, this study is also not free from limitations. A significant limitation of this data set (NFHS-4) is that it gives information about women and men aged 15–49 and 15–54 years, respectively. Hence, any analysis will underestimate the prevalence of diabetes, which is more likely to occur in old age. Another limitation is that it doesn’t distinguish between type 1 and type 2 diabetes.

Another limitation is in the dietary assessment methods, i.e., there might be other food items responsible for the development of diabetes but were not included in the questionnaire of the respondents. Among South Asian countries, starchy food items rich in carbohydrates (more than 60% of their diet consists of carbohydrates) are majorly responsible for the development of diabetes^45^, but this data set lacks information on these variables. Further, data on family history of diabetes and physical activity could have improved the study but the information on the same is not included in the data set. However, we tried to capture a sedentary lifestyle through the frequency of watching TV and physical inactivity through body mass index in this study.

## Policy implications

Gender differential becomes crucial when one has to learn to live with a disease. Women and men lead different life even if they belong to the same socio-economic category, and there are differences in their lifestyles and biological composition. Socio-economic status, dietary habits and lifestyle affect them differently. This study will allow policymakers to focus more on obese, non-vegetarian, richest men and those who follow sedentarism as these factors affect them more. Analysis also says that women who never consume pulses or beans and who consumes egg or chicken or meat daily are being affected by diabetes more than men in the same categories. Urban women and rural men are more diabetic, hence, policymakers should focus on urban and rural areas in a planned way.

Further, study will also allow policymakers to consider the educational status of women and men while coming with measures against diabetes. Among women, who have no schooling are more affected whereas, among men, who have schooling less than 12 years are more affected. Social categories also need to be considered while making policies as different gender is at a different level of risk in various categories. Their needs are different, hence, policies should reflect these differences. This study could be further strengthened by other research capturing adults over 50 years, who are more susceptible to diabetes. Studies incorporating data on family history of diabetes and physical activity will give more accurate picture of diabetes. This study will allow data collecting agencies to incorporate the more effective variables in measuring diabetes and associated risk factors.

## Conclusion

Our findings are important from a policy point of view as it affirms that socio-economic and risk factors play different roles among different genders in diabetes in India. Our findings revealed that women with hypertension are more affected by diabetes than their men counterparts, whereas obese men are at greater risk than obese women. The results also showed that non-vegetarian and vegetarian men are at greater risk than women. In comparison, vegan women are comparatively more affected than men counterparts. Further, men in all wealth quintiles (except richer section) and all social categories are at more risk of diabetes than their women counterparts. It is also observed that sedentary life has a higher impact on diabetes in men than women. Our findings conclude that women and men with similar socio-economic status, biological conditions, and dietary and smoking habits are being affected differently by diabetes. Thus, there is a need for gender dimension in research to understand and validate the differences in the needed interventions for diabetes control in India.
